# Effects of active and sham tDCS on the soleus H-reflex during standing

**DOI:** 10.1007/s00221-023-06624-7

**Published:** 2023-05-05

**Authors:** Lynn M. McCane, Jonathan R. Wolpaw, Aiko K. Thompson

**Affiliations:** 1 Interdisciplinary Neuroscience Program, University of Rhode Island, Kingston, RI 02881 USA; 2 National Center for Adaptive Neurotechnologies, Stratton VAMC, Albany, NY 12208 USA; 3 Department of Health Sciences and Research, College of Health Professions, Medical University of South Carolina, 77 President Street, MSC 700, Charleston, SC 29425 USA

**Keywords:** Spinal reflexes, Neuromodulation, Plasticity, Tibial nerve stimulation

## Abstract

Weak transcranial direct current stimulation (tDCS) is known to affect corticospinal excitability and enhance motor skill acquisition, whereas its effects on spinal reflexes in actively contracting muscles are yet to be established. Thus, in this study, we examined the acute effects of Active and Sham tDCS on the soleus H-reflex during standing. In fourteen adults without known neurological conditions, the soleus H-reflex was repeatedly elicited at just above M-wave threshold throughout 30 min of Active (*N* = 7) or Sham (*N* = 7) 2-mA tDCS over the primary motor cortex in standing. The maximum H-reflex (H_max_) and M-wave (M_max_) were also measured before and immediately after 30 min of tDCS. The soleus H-reflex amplitudes became significantly larger (by 6%) ≈1 min into Active or Sham tDCS and gradually returned toward the pre-tDCS values, on average, within 15 min. With Active tDCS, the amplitude reduction from the initial increase appeared to occur more swiftly than with Sham tDCS. An acute temporary increase in the soleus H-reflex amplitude within the first minute of Active and Sham tDCS found in this study indicates a previously unreported effect of tDCS on the H-reflex excitability. The present study suggests that neurophysiological characterization of Sham tDCS effects is just as important as investigating Active tDCS effects in understanding and defining acute effects of tDCS on the excitability of spinal reflex pathways.

## Introduction

Weak transcranial direct current stimulation (tDCS, 1–4 mA) alters corticospinal excitability (Nitsche and Paulus 2000) and can enhance motor skill acquisition and retention (Devanathan and Madhavan 2016; Buch et al. 2017; Foerster et al. 2018a; Rostami et al. 2020). For example, tDCS over the motor cortex improved a fine motor skill of the hand in healthy participants (Reis et al. 2009), resulted in better balance in older, high fall-risk participants (Yosephi et al. 2018), and improved unique skills of ankle tracking and toe-pinch force tasks in healthy controls (Xiao et al. 2020) and people with stroke (Madhavan et al. 2011; Yamaguchi et al. 2016). At the same time, the mechanisms of tDCS as an adjuvant therapy for enhancing sensorimotor rehabilitation are not well understood. Some have reported that even when tDCS changes corticospinal excitability, it may have no effects on behavioral outcomes (Horvath et al. 2016; Aneksan et al. 2021). Possible explanations for this disconnect between induction of corticospinal plasticity and motor skill improvement (which may or may not be produced by tDCS) may be found in inter-study differences in experimental paradigms, outcome measures, study populations, and individual physiological differences (Ridding and Ziemann 2010; Bikson et al. 2018).

The impact of tDCS on the function and excitability of spinal cord pathways has not been well established. The Hoffmann-reflex (H-reflex), the electrical analog of the spinal stretch reflex, is a measure of spinal reflex excitability. Its amplitude reflects the excitability of its pathway, which is influenced by spinal and supraspinal input (Zehr 2002; Knikou 2008). For example, electrocorticographic activity over the sensorimotor cortex is correlated with H-reflexes in the beta and gamma bands in rodents, and modulation of the electroencephalographic sensorimotor rhythm is related to H-reflex amplitude modulation in people (Boulay et al. 2015; Jarjees and Vuckovic 2016; Thompson et al. 2018). Thus, tDCS that affects cortical activity (Roy et al. 2014; Hordacre et al. 2018) and corticospinal excitability (Nitsche and Paulus 2000; Quiles et al. 2022) may affect the excitability of spinal reflex pathways. However, the effects of tDCS on spinal reflex excitability are not apparent or consistent across currently available literatures. In the lower extremity, a recent study reported that a single-dose of “excitatory” tDCS (anode placed over the leg area of the primary motor cortex M1) increased the submaximal soleus H-reflex amplitude at rest in power athletes (Grospretre et al. 2021). Another study reported that a single-dose of “inhibitory” tDCS (cathode over M1) applied during standing had no effect on the soleus submaximal H-reflexes (Baudry and Duchateau 2014). Yet other studies reported that the soleus maximum H-reflex (H_max_) amplitude was not affected by single-doses of excitatory or inhibitory tDCS in seated individuals (Nitsche et al. 2003; Roche et al. 2011; Grospretre et al. 2021). Studies that examined the effects of tDCS on spinal interneuronal pathways are limited; one study found a single-dose of excitatory tDCS produced no change in reciprocal inhibition of the soleus H-reflex by common peroneal nerve stimulation at the end of the dose (Yamaguchi et al. 2016), while another found it decreased during the dose but not after (Roche et al. 2011). Two studies found presynaptic inhibition of the soleus H-reflex by common peroneal nerve stimulation unchanged after a single-dose of tDCS (Roche et al. 2011; Yamaguchi et al. 2016). In sum, a limited number of studies have examined the effects of tDCS on the excitability of lower extremity spinal cord pathways, and the findings are inconsistent. Again, discrepancies in tDCS applications and/or methods to measure spinal reflex excitability (e.g., in active vs. resting muscles) may have contributed to such variable findings. Thus, in this study, we aimed to examine the effects of tDCS on a spinal pathway that is actively engaged in the simple motor task of standing. Specifically, Active (excitatory) or Sham tDCS was applied during repeated elicitation of the soleus H-reflex while the participant maintained a stable level of soleus EMG activity in standing. The reflex measurements were made before, during, and after 30 min of Active or Sham tDCS. To increase confidence in the findings (in expectation of potentially small effects), the same experiment was repeated four times in each participant.

Spinal reflexes contribute to normal and impaired sensorimotor functions in lower extremity (Zehr and Stein 1999; Thompson and Sinkjaer 2021). Thus, elucidating the impact of tDCS on the excitability a spinal reflex pathway during an active motor task (e.g., standing) is essential for defining its potential therapeutic utility in enhancing lower extremity sensorimotor rehabilitation in neuromuscular disorders.

## Methods

### Participants

Fourteen young adults (9 females and 5 males, mean age = 26.1 years, range 21–36) with no known neurological impairments completed the protocol, which consisted of four sessions of tDCS (Active or Sham) + repeated soleus H-reflex elicitation. Prior to participation, all participants provided written informed consent to the study protocol as approved by the Medical University of South Carolina and the University of Rhode Island Institutional Review Boards and completed a tDCS and transcranial magnetic stimulation (TMS) compatibility screening. They were randomly assigned to the Active (*n* = 7) and Sham (*n* = 7) tDCS groups.

### Study protocol

**Fig. 1:**
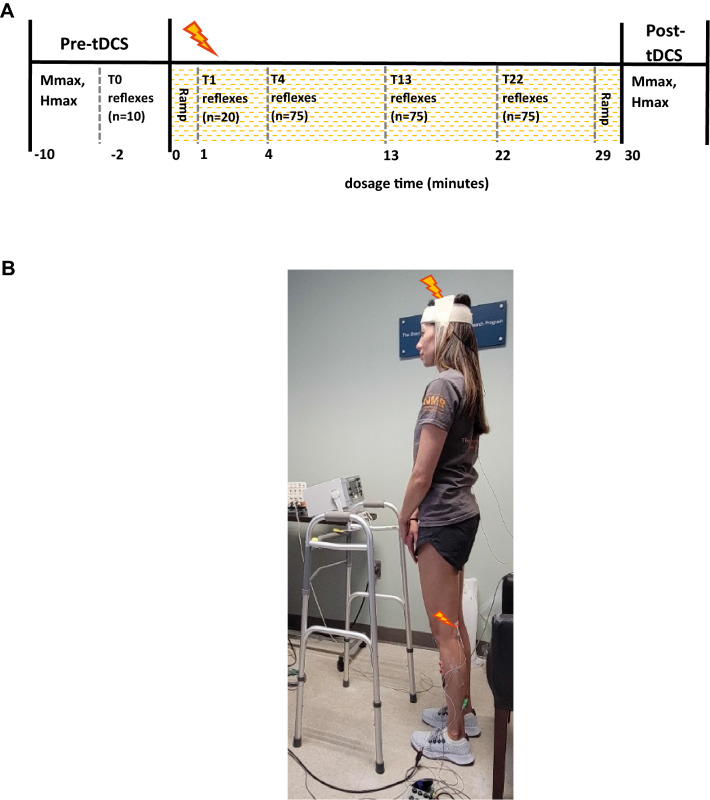
**A** Session protocol. The maximum H-reflex (H_max_) and the maximum M-wave (M_max_) were measured in the soleus before and after 30 min of tDCS. During tDCS, submaximal soleus H-reflexes were repeatedly elicited at a stimulus intensity just above M-wave threshold over four blocks (i.e., T1, 4, 14, and 22). In addition, 10 H-reflex trials were obtained prior to turning on the tDCS (i.e., T0). The same protocol was repeated on four different days. **B** Experimental setup. tDCS and tibial nerve stimulation were delivered while the participant stood and maintained his/her natural standing posture and corresponding level of soleus and TA EMG activity

After a preliminary session, in which the presence of the soleus H-reflex was confirmed, each participant completed four approximately one-hour sessions that were separated by 1–18 days (mean = 3.3 ± 3.6 SD days). Prior to the first experimental session, each participant was randomly assigned to either the Active or Sham tDCS group and remained in the same tDCS condition group for the entire study. The session protocol is summarized in Fig. 1A. At the beginning of each session, EMG recording, tibial nerve stimulation, and tDCS electrodes were placed over the study leg and scalp as optimized during the preliminary session. Then, first, the soleus H_max_ and M_max_ were measured in standing prior to turning on the tDCS. Second, after a 1 min of seated rest, 10 submaximal H-reflex trials were administered in standing while the tDCS remained off (T0). Third, after the T0 (pre-tDCS) block of H-reflex measurement was completed, the participant sat in a chair and the tDCS was turned on. Fourth, when tDCS current had been ramped up to 2 mA, and at least one minute had passed since the T0 reflex block, the participant stood up and received 20 submaximal H-reflex trials (T1). After the T1 block of H-reflex measurement was completed, the participant was seated for at least one minute. Fifth, 225 submaximal H-reflex trials were administered in three blocks of 75 trials each (i.e., T4, T13, and T22 blocks of H-reflexes). At least one minute of seated rest was taken between blocks. These H-reflex trial blocks occurred similarly to the baseline phase of H-reflex operant conditioning studies (Thompson et al. 2009, 2013; Makihara et al. 2014). Upon completing the last block of 75 H-reflex trials (i.e., T22), the participant sat back in the chair and waited until the prescheduled 30 min of tDCS was done, including the full ramps, which was 1 min for Sham (30 s ramp-up to 2 mA, then ramp-down for 30 s) and 30 s for the Active condition. Sixth, when at least one minute had passed since the tDCS was completely turned off, the participant stood up again and the post-tDCS H_max_ and M_max_ measurements were made.

Note that the above blocks of submaximal H-reflex measurements (i.e., T1–T22 blocks) were named according to their specific measurement onset within the 30 min of tDCS; on average, T1 started at 0.9 min, T4 at 4.4 min, T13 at 13.5 min, and T22 at 22.1 min after tDCS was turned on. T0 measurement occurred before the tDCS was turned on.

In addition, to increase confidence in the findings, the same experiment was repeated four times in each participant, and all sessions were held at the same time of day to eliminate a possibility of diurnal rhythm affecting the H-reflex measurements (Wolpaw and Seegal 1984; Chen and Wolpaw 1994; Carp et al. 2006; Lagerquist et al. 2006).

### tDCS

Each participant was randomly assigned to the Active or Sham tDCS group prior to the first experimental session without the participant’s knowledge. They remained in the same group for all 4 study sessions (parallel, single-blinded). The tDCS was applied at the “M1 hotspot”, which was determined for each participant in a separate preliminary session. In the preliminary session, transcranial magnetic stimulation (TMS) was applied to elicit the soleus MEP during standing, using Magstim 200^–2^ and a 110 mm double-cone coil or a custom-made bat-wing coil with radii of 9 cm (Jali Medical Inc., Woburn, MA). The coil was held over the scalp such that the induced current flowed in the posterior–anterior direction in the brain. The hotspot was determined for each individual by systematically moving the TMS coil around the vertex (C_z_) (typically within 0–3 cm lateral and 0–3 cm anterior or posterior to the vertex) at the TMS intensity that produced about a half-maximum MEP (Devanne et al. 1997; Knash et al. 2003; Kido-Thompson and Stein 2004; Thompson et al. 2011) which was estimated at the tentative hot spot (typically 1 cm lateral to the vertex). The minimum interstimulus interval was 5 s. The TMS location that produced the largest soleus MEP through this process was deemed as the hotspot and measured in relation to C_z_ for future sessions. Across all participants, hotspot was found contralateral to the tested leg, typically 2 cm lateral to the vertex.

Bipolar tDCS was delivered using a Soterix MXN-9 stimulator (Soterix Medical Inc., New York, NY) at 2 mA with rubber electrodes encased in 5 × 7 cm saline soaked sponges (0.06 mA/cm^2^ current density, 0.10 C/cm^2^ total charge). The anode was centered over the M1 soleus hotspot, and the cathode was placed over the supraorbital ridge ipsilateral to the EMG recording and nerve stimulation side (Nitsche and Paulus 2000; Patel and Madhavan 2019). The long edge of the anode was placed in the anterior–posterior direction over the hotspot and the cathode placed transversely over the opposite forehead.

For Active tDCS, 30 s of current ramp-up and 30 s of ramp-down were incorporated in the first and thirtieth minutes of the 30 min of tDCS (i.e., 29 min of constant 2 mA stimulation between the ramp-up and ramp-down periods). For Sham tDCS, the current was ramped up to 2 mA over 30 s and then immediately ramped down to 0 mA over the next 30 s, in the first and thirtieth minutes (i.e., 28 min of no stimulation between ramps). These Sham parameters have been shown to have no significant effect on corticospinal excitability while replicating the sensations of Active stimulation (Gandiga et al. 2006; Woods et al. 2016; Dissanayaka et al. 2018). Electrode adjustments were occasionally made based on real-time current delivery reporting on the stimulator to ensure the consistency of tDCS current intensity over 30 min. The tDCS parameters used in this study were within established safety limits (Antal et al. 2017), have been shown to excite the leg motor cortex (Jeffery et al. 2007; Ghosh et al. 2019), and have been used in previous studies in which a spinal reflex was also measured (Bastani and Jaberzadeh 2014; Sriraman et al. 2014; Agboada et al. 2019). Subjective perception of tDCS condition (i.e., Active or Sham tDCS) and sensations were noted in each session for each participant. Every participant reported some sensations at the anode; most reported itchiness and heat, a few reported pinching, and none reported the stimulation as painful. Across all participants from both Active and Sham tDCS groups, 78.6% of the sessions were perceived as Active tDCS sessions (92.8% of Active and 64.3% of Sham participants) when only 50% were Active in reality; two participants with prior tDCS experience also occasionally guessed the condition incorrectly. Thus, we are confident that masking was successful.

### EMG recording and tibial nerve stimulation

EMG signal was recorded from the soleus and its antagonist tibialis anterior (TA) of the leg stimulated. Pairs of self-adhesive surface Ag–AgCl electrodes (2.2 × 3.5 cm, Nissha Medical Technologies, Buffalo, NY) were placed with their centers ~ 3 cm apart. The pair of soleus electrodes were placed just below the gastrocnemii and the TA pair were placed over the muscle belly. EMG signal was amplified and bandpass filtered at 10–1000 Hz using an AMT-8 EMG amplifier system (Bortec Biomedical Ltd., Calgary, AB, Canada), digitized at 3200 Hz, and stored.

To elicit the soleus H-reflex and M-wave, cathodal stimulation was applied to the tibial nerve in the popliteal fossa using disposable surface self-adhesive Ag–AgCl electrodes (2 × 2 cm for the cathode and 2.2 × 3.5 cm for the anode, Nissha Medical Technologies) and a Digitimer DS8R constant current stimulator (Digitimer Limited, Letchworth Garden City, UK). For each participant, the stimulus electrode locations were carefully selected such that the least amount of current was required to elicit the H-reflex in the soleus while minimizing stimulation of the common peroneal nerve and other unwanted nerves. A single 1-ms square pulse was delivered to the tibial nerve stimulating electrode pair when the standing participant had maintained a pre-defined level of soleus (natural standing level) and TA (resting level) EMG activity for at least 2 s, and at least 5 s had passed since the last trial (Hill et al. 2022). To measure the maximum M-wave (M_max_) and H-reflex (H_max_), tibial nerve stimulus intensity was increased in increments of 0.5–3 mA from below soleus H-reflex threshold, to H_max_, to an intensity just above that needed to elicit M_max_ (Thompson et al. 2009; Makihara et al. 2012). At each intensity, four EMG responses were averaged to measure the H-reflex and M-wave; ≈10 different intensities were used to obtain the H_max_ and M_max_ in each participant. For the subsequent submaximal H-reflex trials, an intensity on the rising portion of the recruitment curve was selected for eliciting reflexes, since the H-reflexes at that stimulus level are sensitive to modulatory (i.e., inhibitory or excitatory) input (Crone et al. 1990). The M-wave size that accompanied these H-reflex trials was typically just above threshold level (≈5–10% M_max_) and was maintained throughout the experimental session by occasionally adjusting stimulus current slightly.

At the end of the preliminary session, a thin, soft cast was fitted for each participant’s lower leg to mark the optimal EMG recording and nerve stimulating electrode locations. This cast was used for all subsequent experimental sessions to ensure consistency in electrode placement across multiple sessions.

### Data analysis

A custom MATLAB (MathWorks Inc., Natick, MA) program was used to analyze the EMG data offline. To measure the soleus and TA pre-stimulus (i.e., background: BG) EMG, the EMG signal was full-wave rectified and the mean absolute EMG amplitude was calculated for the 50 ms immediately before stimulation. Soleus H-reflex and M-wave amplitudes were measured as peak-to-peak EMG amplitude, typically in the windows of 32–44 ms post-stimulus for the H-reflex and 6–21 ms post-stimulus for the M-wave.

For analysis of submaximal H-reflexes, the trials with too large or too small M-waves, soleus BG, or TA BG were removed from the statistical analysis to perform adequate comparison of H-reflexes across multiple timepoints. Across all participants, 86 ± 13 SD% of H-reflex trials were included in the final analysis. For each session, soleus, TA, and M-wave values were then normalized to the individual’s pre-tDCS M_max_ for that session. For each participant’s submaximal H-reflexes, H-reflex size at T1, T4, T13, T22 (i.e., H-reflex measurements *during* tDCS), were expressed as a percentage of the T0 value (i.e., pre-tDCS reflex size).

The pre-tDCS and post-tDCS H_max_ were the maximum mean values of four trials from the recruitment curve measurement and expressed as a percentage of the M_max_ that was obtained from the corresponding recruitment curve.

### Statistical analysis

*R* program by the R Foundation for Statistical Computing (https://www.r-project.org) was used for all statistical analysis. First, in expectation of potentially small effects and to verify such small effects, we repeated the same experimental procedures four times in each participant (i.e., on four separate days), and the mean across four sessions was calculated for each measure to test the acute impact of tDCS (+H-reflex elicitation) in standing. Since the number of days between experimental sessions varied within and across participants (mean days between 3.3 ± 3.6 SD, range 1–18 days), the potential effect of repeated tDCS + H-reflex elicitation over multiple sessions was not evaluated further.

To test for significant predictors of soleus and TA BG, M-wave and H-reflex sizes, and change in submaximal H-reflex size from the pre-tDCS block (T0) to the four during-tDCS measurements (i.e., T1, T4, T13, T22), linear mixed models (LMM) that considered the fixed effects of STIM-CONDITION (i.e., Active vs. Sham tDCS) and TIME (i.e., T1, T4, T13, and T22) with participant as the random effect (intercept) were used.

To examine if H_max_ and M_max_ were affected by Active or Sham tDCS, pre- and post-tDCS values were compared using paired *t* test or Wilcoxon Rank-Sum Test (nonparametric data). To assess the stability of EMG and nerve stimulation conditions over the course of the experimental session, the M_max_ amplitude change from pre- to post-tDCS was calculated and expressed in % pre-tDCS M_max_ for each person.

In 3 of 14 participants (one in the Active and two in the Sham tDCS groups), the post-tDCS H_max_ and M_max_ measurements were not available for one session. For those, the values from available sessions were averaged together for further analyses.

For reporting of the results, all mean values are reported with standard deviations (± SD) unless indicated. Significance level was set to *p* = 0.05.

## Results

### Stability of experimental (data collection) condition

The soleus M_max_, which was 11.7 ± 3.8 mV, ranged 5.3–20.1 mV across participants in the pre-tDCS measurement, tended to decrease over an experimental session by a small amount (− 4.3% at post-tDCS, expressed as % of pre-tDCS M_max_), equivalent to ≈0.50 mV change in M_max_. For all participants, the pre- and post- M_max_ values were not significantly different (*p* = 0.60, Wilcoxon paired), nor were they different between the groups (*p* = 0.48, Wilcoxon). The extent of M_max_ change with tDCS + H-reflex elicitation did not differ between the Active or Sham tDCS groups (*p* = 0.62, Wilcoxon test).

Soleus BG EMG (mean of 26 ± 8 μV), TA BG EMG (≈8 μV, corresponding to a resting level) and soleus M-wave size (mean of 0.45 ± 0.02 mV), the stability of which is essential in assessing changes in H-reflex size across multiple measurement time points, remained stable across measurements. For soleus BG, when normalized to the M_max_, the LMM’s total explanatory power was substantial (conditional *R*^2^ = 0.97), and the part related to the fixed effects alone was small (marginal *R*^2^ = 0.04); the effects of STIM-CONDITION (*p* = 0.41), TIME (*p* = 0.40), and their interaction (*p* = 0.70) were not significant. The results were the same when LMM analysis was applied to the raw soleus BG EMG value; the model’s explanatory power was substantial (conditional *R*^2^ = 0.99) and the part related to the fixed effects alone was small (marginal *R*^2^ = 0.008); and the effects of STIM-CONDITION (*p* = 0.76), TIME (*p* = 0.07), and their interaction (*p* = 0.85) were not significant. For TA BG (in raw EMG values), the LMM’s total explanatory power was substantial (conditional *R*^2^ = 0.99), and the part related to the fixed effects alone was small (marginal *R*^2^ = 0.003). The effects of STIM-CONDITION (*p* = 0.84), TIME (*p* = 0.18), and their interaction (*p* = 0.92) did not significantly affect TA BG. For soleus M-wave size that accompanied submaximal H-reflexes, the LMM’s total explanatory power was substantial (conditional *R*^2^ = 0.94), and the part related to the fixed effects alone was small (marginal *R*^2^ = 0.008). The effects of STIM-CONDITION (*p* = 0.70), TIME (*p* = 0.55), and their interaction (*p* = 0.57) did not significantly affect M-wave size. Combined, these results indicate the consistency in reflex measurement condition within experimental sessions.

### Changes in the soleus H-reflex over 30 min of tDCS

**Fig. 2:**
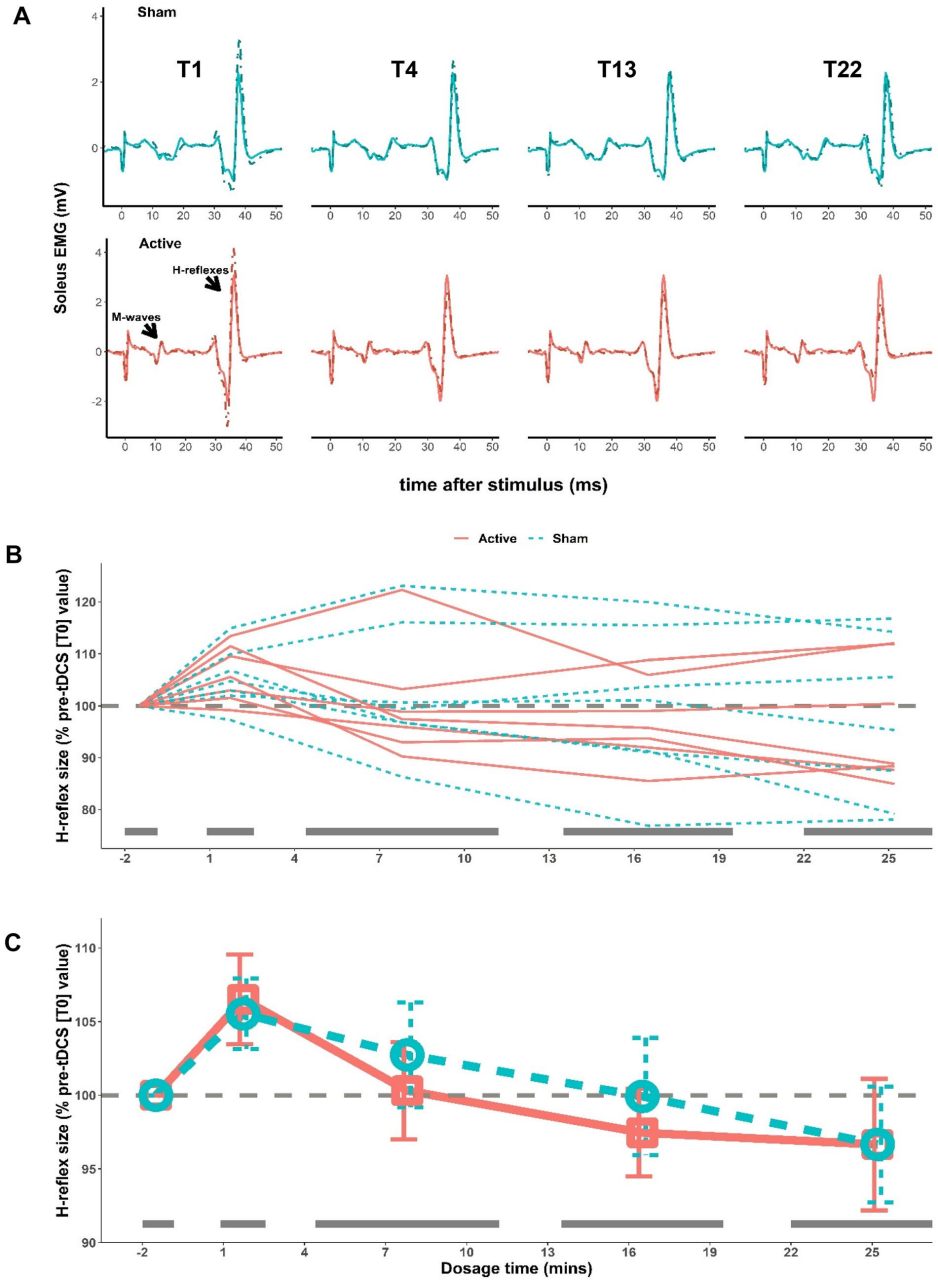
**A** Sample H-reflex traces for a Sham (top) and an Active (bottom) participant in the order of elicitation, from left to right, blocks T1, T4, T13, T22. The solid lines plotted in each graph are a pre-tDCS reflex (T0) for each participant and the dashed lines are a reflex trace from the block collected during tDCS. The increase in the T1 H-reflex size from T0, and its return to pre-tDCS size over time, is easily seen for both participants. **B**, **C** The 4-session mean percent reflex change from T0, y-axis, is plotted over dosage time, x-axis, for each participant in **B** and each group’s block average in** C** (bars are standard error). The dashed gray line at the y-intercept of 100 in **B** and **C** represents no change in reflex size post-tDCS. Sham is cyan dashed lines in **B** and circles in **C**; Active are orange solid lines in **B** and square symbols in **C**. The pre-tDCS block is plotted at − 1.5 min. Post-tDCS blocks are plotted at the midpoint of the tDCS dosage for that block (1.7, 7.8, 16.5, and 25.3 min). Gray bars are mean block durations

To examine if Active or Sham tDCS affected the excitability of the H-reflex pathway over the course of 30 min of tDCS in standing, we fitted a LMM to predict change in submaximal reflex size (expressed as % of the pre-tDCS block, T0) with STIM-CONDITION and TIME. The model's total explanatory power was large (conditional *R*^2^ = 0.79), and the part related to the fixed effects alone was moderate (marginal *R*^2^ = 0.09). The main effect of TIME was significant (*p* = 0.002), while STIM-CONDITION (*p* = 0.89) and the STIM-CONDITION*TIME interaction (*p* = 0.86) were nonsignificant. The temporal change in H-reflex size from T0 was similar in both Active and Sham tDCS groups; there was an initial increase in H-reflex size from the pre-tDCS block (T0) to the first minute(s) (T1) of 6%, then a decrease over the dosage time back to baseline in both groups (Fig. 2). H-reflex size change from T0 to T1–T22 ranged, on average, from 70 to 143% for the Active tDCS group and 59–147% for the Sham tDCS group and the mean change over all post-tDCS blocks was 100 ± 16% for Active and 101 ± 17% for Sham. The same temporal signature of reflex size change was seen in both groups as a temporary, rapid increase in H-reflex size at T1 from T0 followed by, from T4 on, a decrease in H-reflex size back toward pre-tDCS level (Fig. 2). Figure 2 also shows that the mean return to the baseline reflex size appeared to occur sooner for the Active group, at T4, while the Sham group’s return was not until T13. These rapid changes in the soleus H-reflex were clearly visible in individual EMG sweeps (Fig. 2A).

### Effects of tDCS on the soleus H_max_

Across both groups of participants, the H_max_ was 4.8 ± 1.6 mV with a range of 1.3–9.0 mV. To examine if the H_max_ collected during standing was affected by Sham or Active tDCS + submaximal H-reflex elicitation, pre-tDCS H_max_ and post-tDCS H_max_ (both expressed as a % of corresponding M_max_) were compared by paired *t* test. Pre- and post-tDCS H_max_ were not significantly different from each other overall (*p* = 0.99, Wilcoxon) or per group (Active, *p* = 0.53, *t* test; Sham, *p* = 0.97, Wilcoxon). The mean change in the H_max_ was − 3 ± 6% for the Active tDCS group and − 0.7 ± 6% for the Sham tDCS group.

## Discussion

Here, we examined the impact of tDCS delivered during an active motor task on the excitability of a spinal reflex pathway. The size of soleus submaximal H-reflex showed some rapid and temporary changes during 30-min of tDCS over the leg M1. During the first minutes of Active and Sham tDCS, H-reflex size increased, and then within the following several minutes it returned to near pre-tDCS size. The return to baseline appeared to occur more rapidly with Active tDCS. Similar to several previous studies (Nitsche et al. 2003; Roche et al. 2011; Grospretre et al. 2021), the soleus H_max_ that was measured after tDCS was turned off did not differ from the H_max_ measured pre-tDCS, suggesting that there was no immediate offline effect of tDCS on the excitability of this pathway. It is important to note that the changes in submaximal reflex size during Active and Sham tDCS reported here were not seen in other studies that collected multiple soleus H-reflexes with similar timelines but without tDCS (Thompson et al. 2009; Makihara et al. 2014). Below, we discuss the mechanistic and functional implications of these brief tDCS effects on the excitability of a spinal reflex pathway and thereby its behavior.

### Rapid increase in H-reflex size with both Active and Sham tDCS

Unexpectedly, we found with both Active and Sham tDCS, submaximal H-reflexes measured at one minute after tDCS onset (i.e., T1, the measurement started 30 s after the ramp-up was completed for the Active tDCS group and at the end of ramp-down for the Sham tDCS group) were, on average, about 6% larger than the pre-tDCS (i.e., T0) H-reflexes. It is unlikely that this temporary increase in H-reflex size was caused by the soleus H-reflex elicitation/measurement procedures used in the present study. In the previous soleus H-reflex and stretch reflex studies in neurologically normal individuals, there was no systematic time-dependent change in the size of soleus reflexes over the course of 225–245 reflex trials over 30–40 min (Thompson et al. 2009; Makihara et al. 2014; Mrachacz-Kersting et al. 2019); during each of 6 baseline sessions in those studies, no systematic rise or fall of reflex sizes was detected (*N* = 15, 8, and 16 in Thompson, et al. 2009, and Makihara et al. 2014, and Mrachacz-Kersting et al. 2019, respectively). We also analyzed a subset of our existing pool of H-reflex data (without tDCS procedures) from 14 young and healthy individuals (unpublished data); their H-reflex data, sorted to match the T0, T1, T4, and T13 measurement timelines of the present study, indicated no significant effect of time (i.e., H-reflex sizes at T1, T4, and T13 were 103 ± 14, 100 ± 15, and 99 ± 17% of the T0 value). These provide partial support for our interpretation that Active and Sham tDCS procedures could temporarily increase the excitability of soleus H-reflex pathway. This small, rapid increase in reflex size found at T1 dissipated with both Active and Sham tDCS in the following blocks (Fig. 2). Since all H-reflex measures were obtained in the same standing posture with the same background EMG, subthreshold level of changes in the excitability of motoneuron pools or changes in postsynaptic inhibition (e.g., disynaptic reciprocal inhibition) that acts directly onto motoneurons cannot explain these H-reflex changes (Capaday and Stein 1987, 1989; Stein and Capaday 1988). A more probable explanation is that these effects of tDCS were presynaptic, acting at the sensory afferent—motoneuron synapses. That is, presynaptic inhibition over the soleus H-reflex pathway was briefly decreased about a minute into Active and Sham tDCS. In the spinal cord, inhibitory interneurons that presynaptically affect Ia-motoneuron transmission receive excitatory input from cutaneous afferents (mostly indirectly), corticospinal neurons, and other spinal neurons (e.g., other interneurons) (Iles 1996; Pierrot-Deseillingny and Burke 2012), and thus, their excitability decrease could be due to reduction in excitatory input or enhancement in inhibitory input to them. Considering the present experimental paradigm (all measures made in the same static task, posture, and background EMG), increased inhibition or reduced sensory (including cutaneous) afferent excitation of inhibitory interneurons would be an unlikely cause of the temporary reduction of presynaptic inhibition presumably happening then. (Note that, not necessarily a single class of interneurons but potentially different groups of interneurons that exert presynaptic action at the Ia-motoneuron synapses could be involved in the observed temporary changes in the excitability of soleus H-reflex pathway.) In the present paradigm, temporarily decreased corticospinal input to those inhibitory interneurons could be a possible mechanism of the brief H-reflex enhancement, potentially caused by one minute of current injection (as rapid H-reflex changes were observed in both Active and Sham conditions). Such rapid changes in corticospinal excitability with a brief period of tDCS is not surprising; several studies have shown that 4 s of tDCS at 1 mA changes the abductor digiti minimi MEP (Nitsche and Paulus 2000; Nitsche et al. 2005). While the mechanism is as yet unclear, the present findings suggest that one minute of excitatory tDCS (anode over M1) briefly reduces descending influence over presynaptic inhibition from the cortex to the spinal reflex pathway.

### Rapid return of H-reflex excitability to the baseline level

The return (i.e., decrease) of H-reflex size from the initial increase was significant over all participants and appeared to occur sooner among the Active tDCS group participants than the Sham group (Fig. 2). Here, we speculate that this minor but clear difference in the time course of H-reflex size change between the Active and Sham tDCS groups may be a potential reflection of how tDCS works. With Active tDCS, the (probable) initial tDCS effect of reduced excitatory descending input onto presynaptic inhibitory interneurons may have been canceled or replaced by opposing effects during continuous positive current injection via tDCS. The apparent faster decrease in H-reflex size with Active tDCS found in this study may be due to an acute increase in corticospinal excitability caused by excitatory tDCS over leg M1, as reported in several other studies in which motor evoked potentials (MEP) to transcranial magnetic stimulation were measured (Jeffery et al. 2007; Madhavan et al. 2016; Foerster et al. 2018b). With Sham tDCS, the excitatory descending input to spinal inhibitory interneurons returned more gradually to its pre-tDCS level, possibly because there was no *specific* synaptic process that canceled the initial Sham tDCS effect and/or increased corticospinal descending input to inhibitory interneurons.

Interestingly, once the H-reflex decreased back to the pre-tDCS level, with Active tDCS, the continuation of positive current over M1 did not continue to increase the presynaptic inhibition of the soleus H-reflex pathway to decrease reflex size beyond the pre-tDCS level. The absence of further reduction in the Active tDCS group’s H-reflex size from T4 to T22 may be explained by the negotiated equilibrium model of spinal cord plasticity (Wolpaw 2018). In this model, the widely distributed CNS substrates of sensorimotor behaviors (called *heksors*) are in a constant state of negotiation to maintain the key features of their behaviors, toward keeping them satisfactory (Wolpaw and Kamesar 2022). The soleus H-reflex pathway participates in many important behaviors, including standing. Thus, the reduction of its excitability caused by tDCS would likely disturb many heksors that were already functioning well in the young and healthy individuals studied here. It would particularly disturb the heksor responsible for standing, the behavior being performed when tDCS was administered. Responses by the heksor for standing and other heksors may have counteracted or otherwise limited the reduction in H-reflex excitability caused by tDCS, thereby accounting for the lack of further reduction with continued cortical stimulation.

### Implications

The ability of tDCS to induce CNS plasticity, measured with, for example, MEPs, fMRI, EEG, and brain derived neurotrophic factor level (Fritsch et al. 2010; Dayan and Cohen 2011; Stagg et al. 2018), is the basis for its potential therapeutic value. However, the impact of tDCS on spinal reflex pathways is not well defined, despite their known importance for normal motor control and for motor control after CNS injury (Yang et al. 1991; Stein et al. 1993; Zehr and Stein 1999; Sinkjaer et al. 2000; Dietz and Sinkjaer 2007).

In the present study, we observed that the excitability of soleus H-reflex pathway was briefly increased by both Active and Sham tDCS applied during standing, and then, its excitability significantly decreased from this brief enhancement. These temporary changes in reflex excitability were not observed in previous reflex studies in which 225–245 reflex trials were administered over 30–40 min period without tDCS (Thompson, et al. 2009, and Makihara et al 2014, Mrachacz-Kersting et al. 2019). Studies that use the same type of Sham protocol used in this study should be aware of such acute effects of temporary current injection to the cortex. The present findings suggest that understanding and defining the Sham tDCS effects on spinal pathways may be essential when one considers its application in motor control or motor rehabilitation research. The rapid decrease (return) of H-reflex size to pre-tDCS level with Active tDCS suggests that tDCS indeed affects the excitability of spinal reflex pathways, and it is important to further understand the effects of tDCS on spinal pathways for effectively applying tDCS for therapeutic purposes.

Whether the small effects seen here are enough to change a motor behavior is yet to be determined. Many studies already avoid a possible complication shown here with Sham tDCS (i.e., temporary increase of H-reflex excitability) by not beginning the intervention until after the tDCS is turned off, although evidence exists that applying tDCS during practice of novel hand (pinch force) and leg (pattern tracing) motor skills enhance their acquisition (i.e., accuracy and speed) (Reis et al. 2009; Devanathan and Madhavan 2016).

In sum, future clinical studies that use tDCS as an adjuvant therapy should consider the impact of tDCS on the neural pathway of the targeted behavior (if it is known) and carefully evaluate the effects of their Sham procedures.

### Methodological considerations and limitations

Several methodological concerns and limitations in this study warrant discussions. One of the main findings of this study is that during the first minutes of Active and Sham tDCS, H-reflex size increased. Since several prior studies, in which soleus reflexes were repeatedly elicited over the course of 30–40 min, found no systematic changes in soleus reflex size (Thompson et al. 2009; Makihara et al. 2014; Mrachacz-Kersting et al. 2019), we would assume that the presently observed rapid increase in reflex size was due to tDCS. We recognize, however, that this interpretation largely depends on the current choice of sham procedures. Deriving a firm conclusion would require additional tDCS control experiments, such as sham tDCS with zero current injection in naïve participants and delivering current over an “off-target” site (e.g., frontal lobe).

Another methodological concern is that other tDCS montages (other than what we used in this study) may target the leg motor cortex more effectively (Foerster et al. 2018b), and therefore, the findings might have been quite different if other tDCS montages were used. The key mechanism of tDCS is thought to be potentiation of cortical areas related to the target motor behavior, which induces cortical plasticity and improves behavioral outcomes (Bikson et al. 2013; Bortoletto et al. 2015). Currently, how different tDCS dosage parameters (i.e., current density, polarity, electrode montage, duration) may produce different effects on spinal reflex excitability is unknown.

The findings might have also differed if the participants had been walking on a treadmill during tDCS, rather than standing (Fernandez-Lago et al. 2017). They might also have differed in people with CNS injury in whom standing and maintaining balance were more difficult (e.g., difference in functional impact of intervention between neurologically intact individuals and people after spinal cord injury (Thompson et al. 2009, 2022; Makihara et al. 2014)). Further studies are clearly needed to define the utility of tDCS in therapeutic applications.

## Conclusion

This study demonstrated that one minute of Active and Sham tDCS temporarily increased spinal reflex excitability and Active tDCS appeared to enhance its decrease back to pre-tDCS levels. These findings could impact motor behavioral outcomes in studies aiming to enhance plasticity with tDCS. Understanding the effects of Active and Sham tDCS on the CNS pathways that involve the targeted motor behavior is essential for developing tDCS applications that can enhance sensorimotor rehabilitation in people with CNS damage.

## Data Availability

Study data is available upon request.
